# Activation of TAK1 by MYD88 L265P drives malignant B-cell Growth in non-Hodgkin lymphoma

**DOI:** 10.1038/bcj.2014.4

**Published:** 2014-02-14

**Authors:** S M Ansell, L S Hodge, F J Secreto, M Manske, E Braggio, T Price-Troska, S Ziesmer, Y Li, S H Johnson, S N Hart, J-P A Kocher, G Vasmatzis, A Chanan-Kahn, M Gertz, R Fonseca, A Dogan, J R Cerhan, A J Novak

**Affiliations:** 1 Division of Hematology, Mayo Clinic, Rochester, MN, USA; 2 Division of Hematology, Mayo Clinic, Scottsdale, AZ, USA; 3 Division of Biomedical Statistics and Informatics, Mayo Clinic, Rochester, MN, USA; 4 Center for Individualized Medicine, Mayo Clinic, Rochester, MN, USA; 5 Division of Hematology, Mayo Clinic, Jacksonville, FL, USA; 6 Division of Anatomic Pathology and Hematopathology, Mayo Clinic, Rochester, MN, USA; 7 Division of Epidemiology, Mayo Clinic, Rochester, MN, USA

**Keywords:** MYD88, Waldenstrom, lymphoma, TAK1

## Abstract

Massively parallel sequencing analyses have revealed a common mutation within the *MYD88* gene (MYD88_L265P_) occurring at high frequencies in many non-Hodgkin lymphomas (NHLs) including the rare lymphoplasmacytic lymphoma, Waldenström's macroglobulinemia (WM). Using whole-exome sequencing, Sanger sequencing and allele-specific PCR, we validate the initial studies and detect the MYD88_L265P_ mutation in the tumor genome of 97% of WM patients analyzed (*n*=39). Due to the high frequency of MYD88 mutation in WM and other NHL, and its known effects on malignant B-cell survival, therapeutic targeting of MYD88 signaling pathways may be clinically useful. However, we are lacking a thorough characterization of the role of intermediary signaling proteins on the biology of MYD88_L265P_-expressing B cells. We report here that MYD88_L265P_ signaling is constitutively active in both WM and diffuse large B-cell lymphoma cells leading to heightened MYD88_L265P_, IRAK and TRAF6 oligomerization and NF-κB activation. Furthermore, we have identified the signaling protein, TAK1, to be an essential mediator of MYD88_L265P_-driven signaling, cellular proliferation and cytokine secretion in malignant B cells. Our studies highlight the biological significance of MYD88_L265P_ in NHL and reveal TAK1 inhibition to be a potential therapeutic strategy for the treatment of WM and other diseases characterized by MYD88_L265P_.

## Introduction

Somatic mutations in genes whose protein products are involved in cell signaling pathways, apoptosis or inflammatory responses contribute to both the pathogenesis and progression of non-Hodgkin lymphoma (NHL).^1, 2, 3, 4, 5, 6, 7^ Recent analyses of next generation sequencing data have revealed common mutations in the myeloid differentiation factor gene, *MYD88*, in malignant B cells from patients with NHL.^1, 8, 9^ While other mutations in *MYD88* have been reported, by far the most highly conserved variant involves a single base-pair substitution translating into an amino-acid switch from leucine to proline at position 265 (MYD88_L265P_).^10^ In our whole-exome sequencing analysis of 49 diffuse large B-cell lymphoma (DLBCL) tumors, we detected MYD88_L265P_ in 12% of tumors.^1^ MYD88_L265P_ was detected at a higher incidence, 30%, in patients with activated B cell-like DLBCL and at lower levels in patients with primary central nervous system lymphoma, marginal zone lymphoma (MZL), Burkitt's lymphoma, mucosa-associated lymphoid tissue (MALT) lymphoma and chronic lymphocytic leukemia.^8, 10, 11^ The reported prevalence of MYD88_L265P_ appears to be highest in patients diagnosed with Waldenström's macroglobulinemia (WM). Recent studies indicate that up to 100% of WM tumors harbor the MYD88_L265P_ mutation, as do 10–87% of patients diagnosed with IgM monoclonal gammopathy of undetermined significance (IgM-MGUS).^9, 12, 13, 14, 15, 16^ The risk of progression to WM in patients with IgM-MGUS is significantly higher when patients carry the MYD88_L265P_ mutation, strongly implicating MYD88_L265P_ as a driver of this lymphoma and potentially other NHL as well.^16^

MYD88 is an adapter protein that serves to couple Toll-like receptors and IL-1R with downstream signaling intermediates. Specifically, MYD88 is recruited to the cytoplasmic portion of Toll-like receptors and IL-1R leading to the activation of IRAK4 and subsequent phosphorylation of IRAK1, which promotes the oligomerization and activation of TNFR-associated factor-6 (TRAF6).^17^ TRAF6 ultimately recruits TAB2 and activates TAB2-associated TGF-β-activated kinase 1 (TAK1) eventually promoting cell survival through activation of NF-κB.^18^ MYD88_L265P_ yields a constitutively active protein, with initial functional studies in DLBCL cell lines demonstrating that forced overexpression of MYD88_L265P_ confers a selective survival advantage.^10^ This increase in malignant cell survival was associated with enhanced IRAK1 and IRAK4 kinase activity and subsequent downstream NF-κB activation. In WM, culturing cell lines endogenously expressing MYD88_L265P_ with an inhibitor of either MYD88 activation or IRAK1/4 significantly decreased nuclear staining of NF-κB p65, again indicating that MYD88_L265P_ mediates its pro-survival effects through NF-κB signaling.^9^ In addition to NF-κB, both knockdown of MYD88 and use of an IRAK1/4 inhibitor diminished autocrine IL-6 and IL-10 signaling through STAT3 in MYD88_L265P_-overexpressing DLBCL cell lines, indicating that MYD88_L265P_ regulates JAK-STAT3 signaling as well.

Due to the high frequency of MYD88 mutation in WM and other NHL, and its known effects on malignant B-cell survival, therapeutic targeting of MYD88 signaling pathways may be useful clinically. However, while the effects of MYD88_L265P_ on the activity of IRAK1/4 and NF-κB are have been studied previously, we are lacking a thorough characterization of the role of intermediary signaling proteins such as TRAF6 and TAK1 on the biology of MYD88_L265P_-expressing B cells. A better understanding of the proteins involved in MYD88_L265P_ signaling may lead to the development of more targeted and effective therapeutic approaches. Additionally, while the high prevalence and constitutive activation of MYD88_L265P_ suggest that it is a gain-of-function driver mutation for many NHL, especially WM, the presence of other cytogenetic events may also mediate the development and progression of these diseases.

The goal of this project was thus twofold. Firstly, we were interested in investigating the presence of recurring cytogenetic aberrations in WM. To do so, we have performed analyses on both mate-pair and exome sequencing data to identify potential abnormalities in the WM genome at both the chromosomal and gene levels, respectively. These studies, in combination with additional Sanger sequencing and allele-specific PCR, have confirmed the previously reported high prevalence of MYD88_L265P_ in WM. The second aim of this study was to characterize the contribution of intermediary signaling proteins belonging to the NF-κB pathway to the biology of MYD88_L265P_-expressing NHL tumors. To this end, we have analyzed WM and DLBCL cell lines endogenously expressing either wild-type MYD88 or MYD88_L265P_ to examine the interplay between MYD88-mediated activation of TAK1 and cytokine secretion and cellular proliferation of malignant B cells.

## Materials and methods

### Cell lines and patient samples

Tumor samples derived from consenting patients were obtained from the University of Iowa/Mayo Clinic Lymphoma SPORE Biospecimens Core and the Predolin Biobank following approval by the Mayo Clinic Institutional Review Board. OCI-LY19, OCI-Ly7 and SUDHL4 cells were a kind gift from Dr Margaret Shipp (Dana–Farber Cancer Institute). Dr Steve Treon kindly provided the BCWM.1 cells (Dana–Farber Cancer Institute) and the MWCL-1 cells were developed by our laboratory.^19^

### Exome sequencing and analysis

DNA extracted from CD19^+^CD138^+^-sorted cells isolated from the bone marrows of seven WM patients underwent whole-exome sequencing. For five of these samples, CD19^-^CD138^-^ cells were used as paired germline controls to distinguish between acquired somatic aberrations and germline polymorphisms; two patient samples were unpaired. A pipeline developed internally by Mayo Clinic was used for the analysis, and the methods have been included in the Supplementary Materials.

### Mate-pair sequencing, analysis and validation

DNA was extracted from CD19^+^CD138^+^-sorted cells isolated from the bone marrows of 15 WM patients (two samples were derived at different time points from the same patient and analyzed separately) using the Gentra PureGene DNA Isolation Kit (Qiagen, Valencia, CA, USA). To serve as a control, germline DNA was extracted from the CD19^−^CD138^−^ cells that remained after enriching for the malignant B cells of three patients. Construction and sequencing of the mate-pair libraries was performed as described previously, and mapping of the mate-pair reads to the Hg19 reference genome was facilitated using novel algorithms developed by Mayo Clinic.^20, 21^

To validate predicted chromosomal events in single patients, primer sets were designed specifically for each abnormality using Primer-Blast (http://www.ncbi.nlm.nih.gov/tools/primer-blast/). Genomic DNA from the affected patient as well as from unaffected controls (100 ng) was amplified via PCR using HotStar Taq DNA polymerase (Qiagen) and a GeneAmp PCR System 9700 (Applied Biosystems, Grand Island, NY, USA) over 35 cycles (95C × 30 s, 55C × 30 s, 72C × 3 min). After amplification, products were run on a 1% agarose gel and visualized under UV light. The post-PCR products were then sequenced using the same primers used for PCR amplification. Sequences were analyzed using Sequencher Version 5.0.1 (GeneCodes, Ann Arbor, MI, USA) and aligned to the human genome using BLAST (http://blast.ncbi.nlm.nih.gov/Blast.cgi).

Predicted copy number abnormalities greater than 10 Mb in size were determined, as previously described, by plotting the number of mate pairs mapping within a given window (W 9–11 kB) against the position on each chromosome.^21^ Variance from the number of mate pairs expected for a copy-neutral locus was indicative of a copy number gain or loss at that position.

### Detection of MYD88_L265P_

DNA or RNA from normal controls (immune tissue, *n*=5) or NHL tumors (WM *n*=32, lymphoplasmacytic lymphoma *n*=5; splenic marginal zone lymphoma *n*=35; nodal marginal zone lymphoma *n*=21 or mucosal-associated lymphoid tissue (MALT) *n*=23) was purified and amplified by PCR. The PCR fragments were sequenced at the Mayo Clinic DNA Sequencing Core. Analysis was done using Mutation Surveyor software (Softgenetics, State College, PA, USA). Full details are described in the Supplementary material. Real-time allele-specific oligonucleotide PCR was performed on available WM DNA using qBiomarker Somatic Mutation Assay for MYD88_85940 (SABiosciences, Qiagene, Hilden, Germany) according to the manufacturer's protocol. The RT-PCR reaction was analyzed using CFX96 real-time thermal cycler (Bio-Rad, Hercules, CA, USA). To obtain a DDC_t_ range for wild-type alleles, the assay was performed on DNA from 10 MYD88_WT_ controls. The cut-off for wild-type versus mutant MYD88 was a ΔΔC_t_ value of 0.002.

### Immunoprecipitation and immunoblotting

B-cell lysates were immunoprecipitated with anti-IRAK1, IRAK4 or TAK1 bound to Dynabeads protein A (Invitrogen Life Technologies, Grand Island, NY, USA). HA-tagged MYD88_WT_ and MYD88_L265P_ cell lysates were immunoprecipitated using anti-HA magnetic beads following the manufacturer's instructions (Pierce, Rockford, IL, USA), then separated by SDS–PAGE and transferred to polyvinylidene fluoride membranes. Membranes were analyzed by immunoblot for MYD88 (Santa Cruz, Santa Cruz, CA, USA), TRAF6 (Abcam, Cambridge, MA, USA), pTAK1, TAB2, IRAK1, IRAK4, TAK1 (Cell Signaling, Danvers, MA, USA) or HA (Roche, Indianapolis, IN, USA).

### Lentiviral production and infection

HA-tagged MYD88_WT_ and MYD88_L265P_ were PCR-amplified and cloned into pLEX-MCS (Open BioSystems, Thermo Scientific, Pittsburgh, PA, USA). Sequence confirmation was obtained by Sanger sequencing. Lentiviral particles were generated using the calcium phosphate transfection reagent kit following the manufacturer's instructions (Open Biosystems, Thermo Scientific). Approximately 50 000 IFU/ml lentiviral particles were then used to infect HEK293T cells, and the cells were selected in puromycin (200 ng/ml) before use.

### NF-κB reporter assay

HEK293 cells expressing a vector control or WT and L265P MYD88 were transiently transfected with 1 ng Renilla and 10 μg of a pNF-κB-luciferase reporter plasmid. After 16 h, luciferase activity was measured in cell extracts and normalized against Renilla with the Dual Luciferase Kit (Promega, Madison, WI, USA).

### Proliferation and apoptosis assays

OCI-Ly3, BCWM.1, MWCL-1 or WM patient cells (0.25 × 10^5^ cells/well) were cultured in triplicate in 96-well flat-bottom microtiter plates at 37 °C in the presence of 5% CO_2_ and 0–10 μM TAK1 (5Z-Oxozeaenol 3604, TOCRIS Bioscience, Minneapolis, MN, USA), IRAK1/4 (Sigma-Aldrich, St Louis, MO, USA) or NF-κB (BAY-11–7082, Cayman Chemical, Ann Arbor, MI, USA) inhibitors. After 48 h, cells were pulsed with 0.05 mCi tritiated thymidine (Amersham, Piscataway, NJ, USA) for 18 h. Cells were incubated with 0.1% Triton X-100, and ^3^H-TdR incorporation levels were determined using a MicroBeta TriLux (PerkinElmer, Waltham, MA, USA). All cell line proliferation studies were independently repeated three times. WM patient cells were treated with 0–10 μM TAK1 inhibitor and after 48 h, cells were incubated with Annexin-V-FITC (Invitrogen; Carlsbad, CA, USA) for 15 min, washed in ice-cold buffer and stained with 1.0 μg propidium iodide. Samples were immediately analyzed on a FACSCalibur flow cytometer and data analysis performed with Cell Quest software (Becton Dickinson; San Jose, CA, USA). Viability was defined as the percentage of total cells that were negative for both Annexin-V and propidium iodide.

### Measurement of IL-10

Cells were cultured for 3 days with the indicated inhibitors, and supernatants were analyzed with the Human Ultrasensitive Cytokine 10-Plex Panel (Life Technologies). Samples were run in duplicate, and the assay was performed according the manufacturer's instructions. Plates were read on a Luminex-200 system Version 1.7 (Luminex, Austin, TX, USA) and analyzed using Star Station software (Applied Cytometry, Sheffield, UK).

## Results

### Exome and mate-pair sequencing analysis

A full description of all WM patient characteristics, tissues analyzed and analytical methods is included in Supplementary Table 1. The exome of five tumor-normal pairs and two tumor-only samples were sequenced and analyzed for recurrent single nucleotide variations (SNVs) and insertions or deletions. Greater than 96.6% of reads were mapped for all 12 samples (paired and unpaired), with 80–90% coverage across target regions at a depth of 30X. The only conserved somatic coding SNV detected in more than one patient and predicted to have a significant impact on protein function was the previously identified mutation in *MYD88* on chromosome 3. This mutation (position 38182641, T→C) results in the substitution of a proline residue for a leucine at amino-acid position 265 (MYD88_L265P_).^9^ MYD88_L265P_ was detected in four of the five paired samples and both of the tumor-only samples for a total of six of seven samples. A gene-level analysis revealed two samples with mutations in *SPAG17*. However, the two SNVs were far apart from one another and not predicted to have functional impact. No recurring insertions or deletions were found, although one patient had two insertions or deletions in *GNPAT.* A list of all of the potentially functional coding SNVs (non-sense, frame-shifting, splice site and missense) detected in the five paired samples is provided in Supplementary Table 2.

As the tumor microenvironment is thought to play a strong role in driving the progression of WM, we also investigated whether the five patient-matched CD19^-^CD138^-^ bone marrow cells used as controls in this study were defined by any mutations not present in a cohort of more than 1000 controls previously sequenced by Mayo Clinic for genetic studies of other disease states. However, no recurring mutations in the five control samples were detected after filtering against the larger Mayo database.

Mate-pair sequencing and analysis revealed at least one predicted chromosomal rearrangement in all of the fifteen patients included in the study. However, all events occurred in single patient samples. Large copy number abnormalities were predicted in eight of sixteen samples, including four copy number abnormalities involving the partial or complete loss of 6q, as identified previously in WM (Supplementary Table 3 and Supplementary Figure 1).^22, 23, 24^ Our data are also consistent with published studies of WM patients reporting common losses in chromosomes 11, 13 and 17 and gains in chromosome 3.^14, 22, 24^

### Validation of MYD88_L265P_

**Figure 1:**
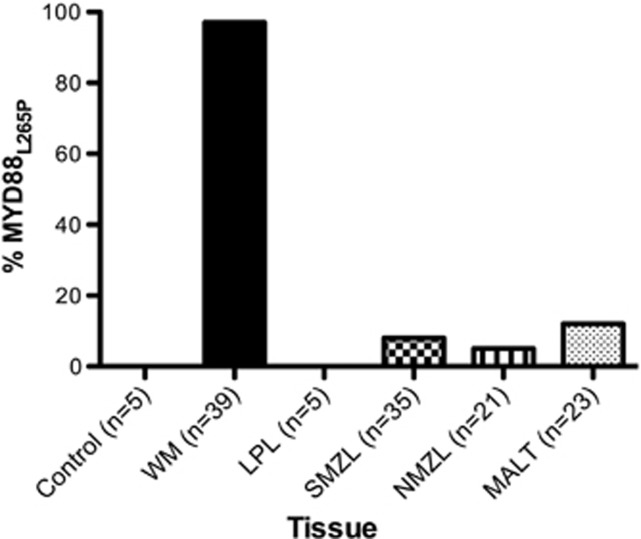
MYD88_L265P_ expression rates in NHL. The MYD88_L265P_ mutation status in biopsies from normal controls, WM, LPL, splenic marginal zone lymphoma, nodal marginal zone lymphoma and MALT lymphomas was determined by exome sequencing, Sanger sequencing or allele-specific PCR rates as described in Materials and Methods. LPL, lymphoplasmacytic lymphoma.

Using Sanger sequencing or real-time quantitative PCR, we confirmed the high prevalence of MYD88_L265P_ in WM. When combined with the exome analysis, we detected MYD88_L265P_ in 97% of WM cases (Figure 1). MYD88_L265P_ was detected at lower frequencies in other indolent lymphomas including lymphoplasmacytic lymphoma (0%), MALT (4%), nodal MZL (5%) and splenic MZL (8%); all but one MYD88_L265P_ was heterozygous. In addition, the WM cell line MWCL-1 and the IgM-secreting cell line BCWM.1 also expressed heterozygous MYD88_L265P_.

### MYD88_L265P_ drives activation of TAK1

**Figure 2:**
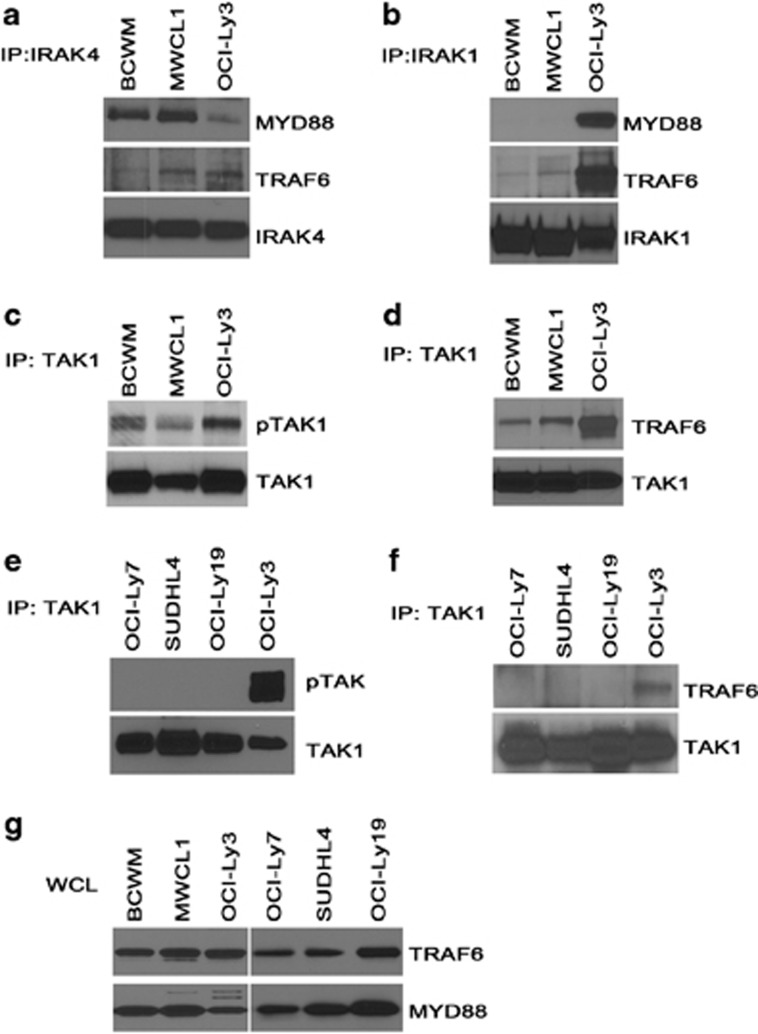
Activation of the TAK1 pathway in WM and DLBCL cells by MYD88_L265P_. (**a**) IRAK4 or (**b**) IRAK1 was immunoprecipitated from BCWM, MWCL-1 or OCI-Ly3 cell lines and immunoblotted for MYD88, TRAF6, IRAK1 or IRAK4. TAK1 was immunoprecipitated from MYD88_L265P_ (**c** and **d**) or MYD88_WT_ (**e** and **f**) cells and analyzed by immunoblot for pTAK1 or TRAF6. Total TAK1 is shown in the lower panel of each figure. (**g**) Whole-cell lysates from the above cell lines were analyzed for expression of MYD88 and TRAF6. All experiments were reproduced a minimum of three times with a representative experiment shown.

In DLBCL tumors, it is known that MYD88_L265P_ promotes cell survival through its spontaneous assembly into a complex with IRAK4 and IRAK1, leading to IRAK1 phosphorylation and NF-κB activation.^10^ However, the correlation between MYD88_L265P_ and the activation of downstream signaling pathways or cell growth and survival has not been investigated in WM. To address this, we explored the possibility that MYD88 signaling pathways are constitutively active in WM cells expressing MYD88_L265P_. Using three cell lines expressing MYD88_L265P_ (BCWM.1, MWCL-1 and OCI-Ly3), we monitored the formation of a complex comprised of MYD88, IRAK1, IRAK4 and TRAF6. Immunoprecipitation of either endogenous IRAK4 (Figure 2a) or IRAK1 (Figure 2b), revealed constitutive association of IRAK with TRAF6 and MYD88_L265P_. In the DLBCL cell line OCI-Ly3, which carries a homozygous mutation in MYD88_L265P,_ we detected high levels of IRAK1 associated with MYD88_L265P_ and TRAF6. However in the WM cell lines, BCWM.1 and MWCL-1, we detected higher levels of IRAK4 associated with MYD88_L265P_. To assess if the formation of a MYD88_L265P_/IRAK/TRAF6 complex results in downstream activation of TAK1, constitutive TAK1 phosphorylation was monitored and detected in all three cell lines (Figure 2c). An association between TAK1 and TRAF6, another measure of TAK1 activation, was also detectable (Figure 2d). When a similar analysis of TAK1 was performed in DLBCL cells expressing MYD88_WT_ (OCI-Ly7, SUDHL4, OCI-Ly19), no phosphorylation of TAK1 (Figure 2e) was detected, nor was TAK1 associated with TRAF6 (Figure 2f). IRAK1, IRAK4, TAK1, TRAF6 and MYD88 were expressed at similar levels in all cell lines studied (Figure 2a–c and g). Therefore, differences in baseline protein expression do not contribute to the differences in MYD88 complex formation observed between cell lines. Taken together, these data suggest that MYD88_L265P_, but not MYD88_WT_, constitutively forms a complex with IRAK and TRAF6 resulting in activation of TAK1 in both WM and DLBCL cell lines.

**Figure 3:**
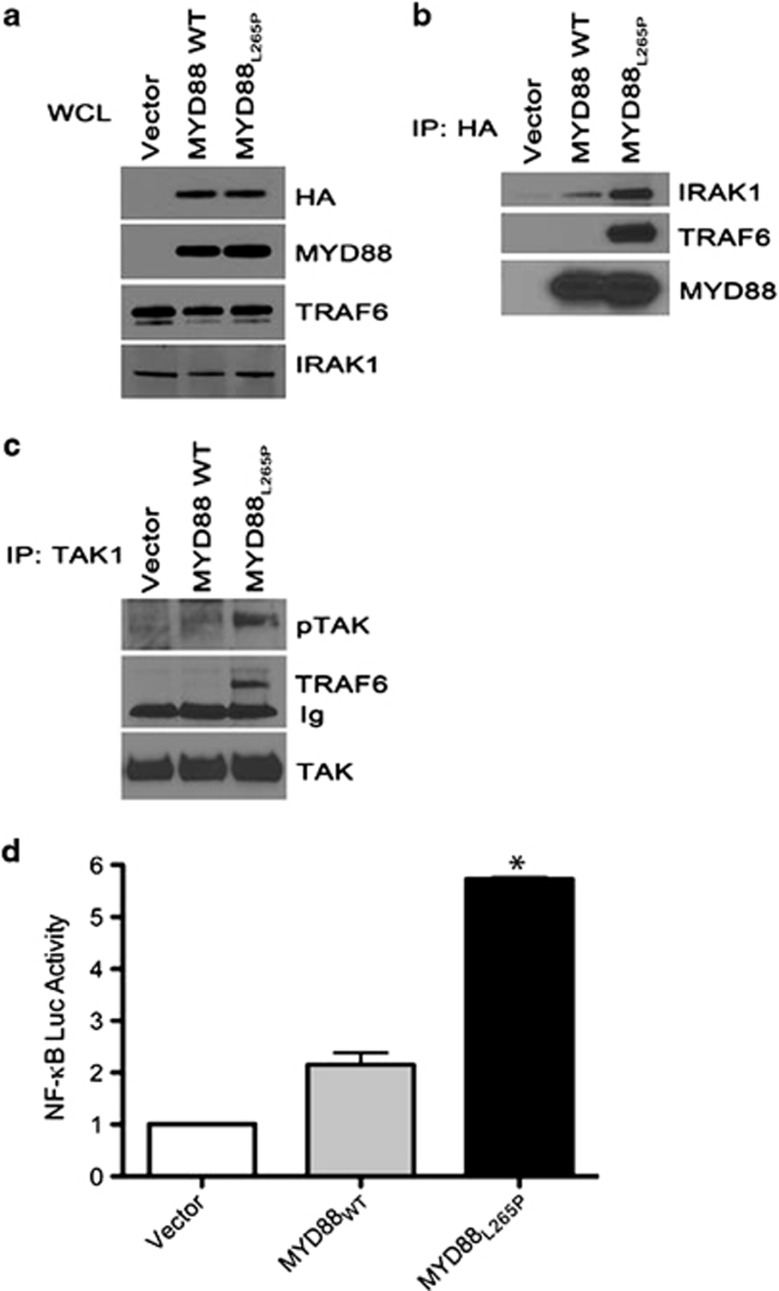
Validation of TAK1 activation by MYD88_L265P_. (**a**) HEK 293T cells were transduced with a vector control or HA-tagged MYD88_WT_ and MYD88_L265P_ expression plasmids. Western blot analysis for HA, MYD88, TRAF6 and IRAK1 was performed on whole-cell lysates from each cell line. (**b**) HA-tagged MYD88 was immunoprecipitated from vector control, MYD88_WT_, and MYD88_L265P_-expressing cells and immunoblotted for IRAK1, TRAF6 and MYD88. (**c**) TAK1 was immunoprecipitated from vector control, MYD88_WT_ and MYD88_L265P_-expressing cells and analyzed by immunoblot for pTAK1 or TRAF6. Total TAK1 is shown in the lower panel of each figure. (**d**) NF-κB luciferase reporter assay in cells expressing vector control, MYD88_WT_ or MYD88_L265P_ . All experiments were reproduced a minimum of three times with a representative experiment shown. *Denotes a *P*-value of <0.05 compared with MYD88_WT_, analyzed by Student's *t*-test.

Comparisons between cells expressing MYD88_WT_ and MYD88_L265P_ clearly suggest that MYD88_L265P_ constitutively drives intracellular signaling. However, some of these differences in pathway activation may be due in part to genetic variation between cell lines. Therefore, a cell line model was generated allowing for a direct comparison of the activation of signaling pathways between MYD88_WT_ and MYD88_L265P_ in a common genetic background. HEK 293T cells were transduced with either a vector control plasmid or HA-tagged MYD88_WT_ or MYD88_L265P_ expression plasmids. Western blot analysis on whole-cell lysates revealed expression of HA-tagged MYD88_WT_ or MYD88_L265P_ in the MYD88-transduced cell lines but not in the vector control. TRAF6 and IRAK1 were detected in all three transduced cell lines at similar levels (Figure 3a). Following HA-immunoprecipitation, an increased amount of IRAK1 and TRAF6 was found to be associated with MYD88_L265P_ as compared with MYD88_WT_ (Figure 3b). Immunoprecipitation of TAK1 revealed increased TAK1 phosphorylation and TRAF6 association in MYD88_L265P_-expressing cells suggesting that TAK1 is constitutively activated in following forced expression of MYD88_L265P_ (Figure 3c). The impact of MYD88_L265P_ on the activation of NF-κB was also measured. Using an NF-κB luciferase reporter, we observed that cells expressing MYD88_L265P_ had a significant increase (*P*<0.01) in NF-κB activation as compared with MYD88_WT_ (Figure 3d). Together, these studies suggest that MYD88_L265P_ forms a complex with IRAK and TRAF6 resulting in constitutive activation of TAK1 and NF-κB.

### TAK1-dependent proliferation and cytokine secretion by myd88_l265p_-expressing cells

**Figure 4:**
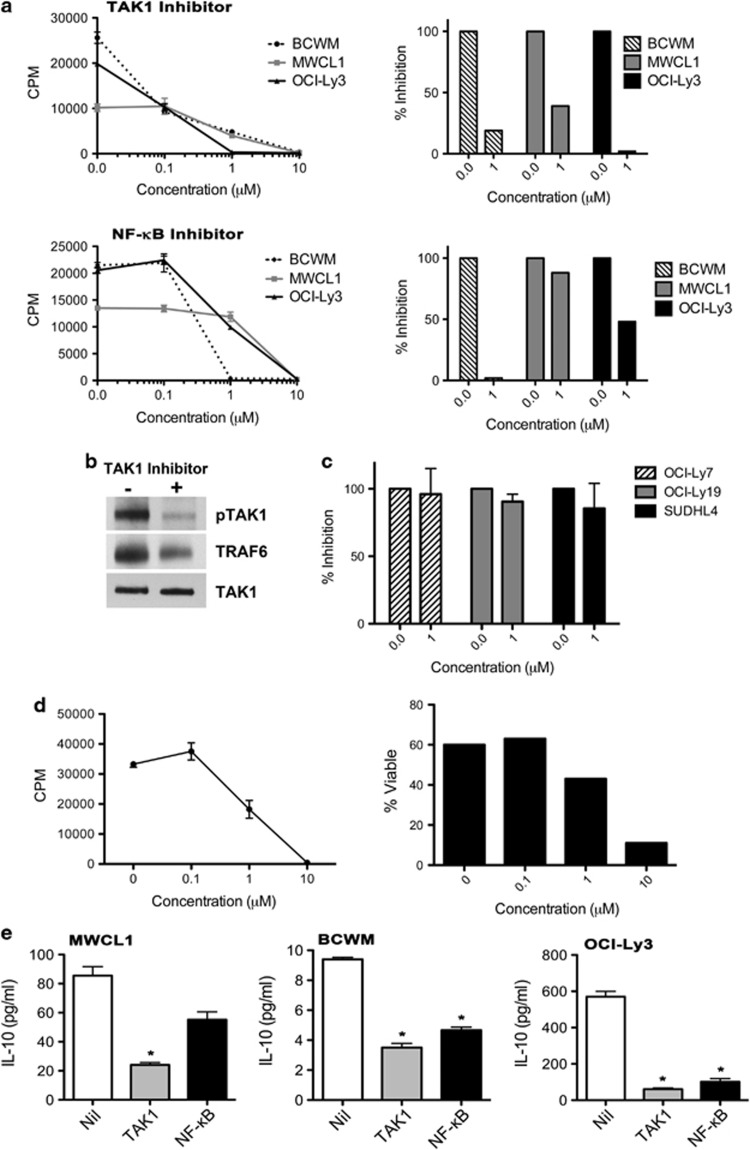
Inhibition of TAK1 decreases cell proliferation and cytokines secretion. (**a**) Proliferation of BCWM, MWCL-1 or OCI-Ly3 cell lines was analyzed in the absence or presence of the TAK1 or NF-κB inhibitors (0.1–10 μM). The data from all drug doses are shown in the left panel, and the percent inhibition at the indicated dose is shown in the right panel. For this analysis, these data is normalized to the nil control for each cell line. The assay was performed in triplicate and a representative experiment is shown (*n*=3). (**b**) TAK1 was immunoprecipitated from OCI-Ly3 cells treated with (+) or without (−) 1 μM TAK1 inhibitor and analyzed by immunoblot for pTAK1 or TRAF6. Total TAK1 is shown in the lower panel. (**c**) Proliferation of OCI-Ly7, OCI-Ly19 or SUDHL4 cell lines was analyzed in the absence or presence of the TAK1 inhibitor (1 μM). (**d**) Proliferation and viability of WM patient cells was analyzed in the absence or presence of the TAK1 inhibitor (0–10 μM). (**e**) Expression of IL-10 by BCWM, MWCL-1 or OCI-Ly3 cell lines was analyzed in the absence or presence of the TAK1 or NF-κB (1 μM) inhibitors as described in Materials and Methods. The assay was performed in duplicate and a representative experiment is shown (*n*=2). *Denotes a *P*-value of <0.05 compared with the nil control analyzed by Student's *t*-test.

To confirm the significance of TAK1-mediated MYD88_L265P_ signaling on lymphoma cell growth, the effect of the selective TAK1 inhibitor, (5Z)-7-Oxozeaenol, on cell proliferation was tested; an NF-κB inhibitor was included as a control (Figure 4a).^25^ All MYD88_L265P_-expressing cell lines were sensitive to TAK1 and NF-κB inhibition in a dose-dependent manner (0–10 μM) (Figure 4a). The percent inhibition of proliferation at the 1 μM of (5Z)-7-Oxozeaenol is a shown graphically on the right side of the panel. To ensure that (5Z)-7-Oxozeaenol inhibited TAK1, OCI-Ly3 cells were treated with 1 μM (5Z)-7-Oxozeaenol for 4 h, TAK1 was immunoprecipitated, and pTAK levels and TRAF6 association were measured (Figure 4b). In the presence of the TAK1 inhibitor, there was a 73% reduction in TAK1 phosphorylation and a 41% reduction in the level of TRAF6 associated with TAK1. NHL cells expressing MYD88_WT_ were found to be insensitive to TAK1 inhibition (Figure 4c). We next tested the impact of the TAK1 inhibitor on a MYD88_L265P_-positive WM patient sample. Similar to what was seen in the WM cell lines, the TAK1 inhibitor inhibited WM cell growth and survival in a dose-dependent manner (Figure 4d).

The MYD88 pathway drives the production and secretion of cytokines, many of which, including IL-10, are known to be involved in the regulation of malignant B cells.^26, 27^ Therefore, the impact of TAK1 inhibition on the expression of IL-10 was investigated, with an NF-κB inhibitor used as a control. At concentrations observed to inhibit cell proliferation, but not impact survival, the TAK1 inhibitor significantly reduced (*P*⩽0.01) the level of IL-10 secreted by each of the cell lines (Figure 4e). A similar trend was seen with IL-6 (data not shown). Together, these data suggest that MYD88_L265P_ drives cell proliferation and cytokine secretion through a TAK1-dependent mechanism.

## Discussion

Through a combination of exome sequencing, Sanger sequencing and Real-time allele-specific oligonucleotide PCR, we have confirmed, in a cohort of 39 patients, the high prevalence of MYD88_L265P_ mutation in WM.^9, 14, 28^ Consistent with other reports, 97% of our patient samples were identified to harbor MYD88_L265P_, with all but one patient being heterozygous.^11, 13, 27^ Frequencies of MYD88_L265P_ were significantly lower in the other low-grade lymphomas analyzed, including splenic MZL, nodal MZL and MALT; MYD88_L265P_ was completely absent in the five lymphoplasmacytic lymphoma patients included in this study. Furthermore, neither the exome sequencing nor an additional mate-pair analysis revealed additional novel common genetic events in our WM patient population. Taken together, our data add to a growing body of literature^29^ underscoring the significance of MYD88_L265P_ as a major driver of pathogenesis in this malignancy.

Initial studies have reported substantial decreases in cellular proliferation and apoptosis following MYD88 inhibition in WM, an effect occurring subsequent to the downstream inactivation of NF-κB signaling.^9, 14^ However, a thorough characterization of the actual signaling intermediates utilized by MYD88_L265P_ to regulate NF-κB in WM is lacking. This knowledge would not only provide a better understanding of the biological role of MYD88_L265P_ in WM tumors, but may reveal new therapeutic targets for the treatment of this and other MYD88_L265P_-driven diseases. To this end, we focused our subsequent investigations on the interplay between mutant MYD88 and TAK1. When activated downstream of MYD88, TAK1 triggers NF-κB signaling, and, in both healthy and malignant B cells, has been associated with regulating proliferation, survival and cytokine secretion.^30, 31, 32^

More specifically, TAK1 is phosphorylated following IRAK-mediated activation of TRAF6. As reported previously by Ngo *et al.,*^10^ we detected constitutive association of both IRAK1 and IRAK4 with TRAF6 and MYD88 in the MYD88_L265P_-expressing DLBCL cell line, OCI-LY3. However, in the WM cell lines, MWCL-1 and BCWM.1, we detected baseline complex formation of only IRAK4, but not IRAK1, with TRAF6 and MYD88. We hypothesize that the differential association of MYD88 and TRAF6 in with IRAK1 versus IRAK4 in DLBCL cell lines may be due in part to the homozygous versus heterozygous nature of the MYD88_L265P_ mutation in OCI-LY3 cells as compared with either MWCL-1 or BCWM.1, respectively. It is possible that baseline activation of MYD88_L265P_ signaling is susceptible to a gene-dose effect, such that cells harboring two copies of the mutated gene, such as OCI-LY3, may actually exhibit enhanced constitutive signaling, and hence detectable complex formation of MYD88 with both IRAK1 and IRAK4 at baseline, as compared with cells expressing only one copy of the L265P mutation. To further support this hypothesis, constitutive formation of the MYD88_L265P_/IRAK/TRAF6 complex was associated with downstream baseline phosphorylation of TAK1 in all three cell lines, but the levels TAK1 phosphorylation was highest in the MYD88_L265P_-homozygous OCI-LY3 cells. In three MYD88_WT_-expressing DLBCL cell lines used as a control, neither baseline MYD88/IRAK/TRAF6 complex formation nor constitutive downstream TAK1 phosphorylation was detected, suggesting that MYD88_L265P_ but not MYD88_WT_ is associated with constitutive activation of intracellular signaling through TAK1. Similarly, constitutive MYD88/IRAK/TRAF6 complex formation and subsequent phosphorylation of TAK1 was associated with nearly threefold higher levels of baseline NF-κB activation in cells overexpressing MYD88_L265P_ as compared with MYD88_WT_.

As TAK1 appears to play an integral role in mediating constitutive MYD88_L265P_ activation of NF-κB in both WM and DLBCL, it is not surprising that use of a TAK1-specific inhibitor significantly decreased cell proliferation and viability. In fact, inhibition of TAK1 in mantle cell lymphoma had a more pronounced effect on cellular proliferation than the NF-κB inhibitor used as a positive control, more than likely due to the fact that TAK1 impacts the activity of other survival regulators in addition to NF-κB, including p38 and XIAP.^32^ These data suggest that while NF-κB appears to be the ultimate driver of abnormal B-cell activity in MYD88_L265P_-expressing cells, targeting MYD88 signaling further upstream of NF-κB may be more effective clinically.

In addition to enhancing baseline NF-κB activation, MYD88_L265P_ also promotes malignant B-cell growth by promoting autocrine and paracrine signaling of cytokines through activation of the JAK-STAT pathway in DLBCL cells. Knockdown of MYD88, as well as inhibition of IRAK, was associated with decreases in both IL-6 and IL-10.^10^ We report here that use of a TAK1 inhibitor also significantly decreases the autocrine secretion of these two cytokines from WM cell lines. Malignant WM B cells are known to be highly under the influence of circulating cytokines. Specifically, IL-6 has been detected at significantly higher levels in both the serum and bone marrow of patients with WM than in non-malignant controls and is associated with increases in IgM secretion.^27^ While the role of IL-10 has not been specifically examined in WM, elevated levels of this cytokine have been detected in many lymphomas and are associated with shorter event-free survival.^26^ Taken together, these results suggest inhibitors of TAK1 may be effective therapeutically against MYD88_L265P_-expressing tumors due to their ability to suppress not one, but several molecular mechanisms critical to the survival of malignant B cells.

In conclusion, we are the first to validate by next generation sequencing in a large patient cohort the high prevalence and specificity of MYD88_L265P_ in WM. Cells harboring the L265P mutation but not wild-type MYD88 exhibit constitutive signaling leading to the hyperactivation of NF-κB. We have established the role of TAK1 as an integral component of MYD88_L265P_ signaling in both WM and DLBCL cell lines, and have identified multiple pharmacodynamic mechanisms by which TAK1 inhibitors may limit the growth and survival of malignant B cells. Our data suggest that targeting TAK1 clinically may be an effective strategy for the treatment of WM and other lymphomas driven by MYD88_L265P_ signaling.
