# Supplementary figures and images for: Activation of TAK1 by MYD88 L265P drives malignant B-cell Growth in non-Hodgkin lymphoma
Source: Blood Cancer J. 2014 Feb 14;4(2):e183–. doi: 10.1038/bcj.2014.4 (PMC3944662; doi:10.1038/bcj.2014.4)

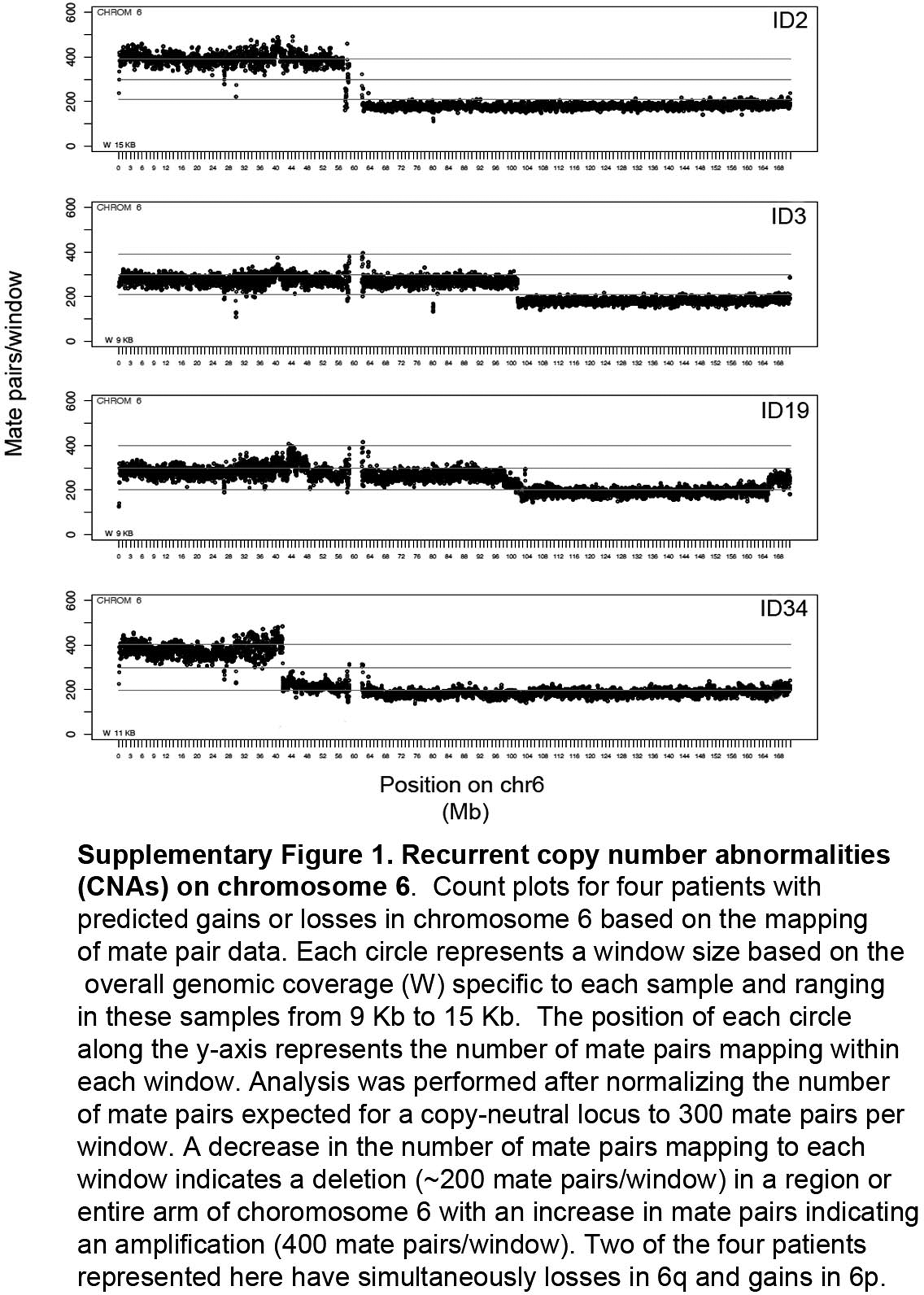

Supplement: Supplementary Figure 1 [file bcj20144x1.tif]
