# Supplementary material for: Activation of TAK1 by MYD88 L265P drives malignant B-cell Growth in non-Hodgkin lymphoma
Source: Blood Cancer J. 2014 Feb 14;4(2):e183–. doi: 10.1038/bcj.2014.4 (PMC3944662; doi:10.1038/bcj.2014.4)
Supplement: Supplementary Table 1 [file bcj20144x2.pdf]

Supplementary Table 1. Waldenstrom patient characteristics and methodology used for $MYD88_{L265P}$ analysis.

Patient Characteristics						Tissue Analyzed			Method Used to Detect $MYD88_{L265P}$				
**ID #**	**Diagnosis**	**Sex**	**Age**	**IgM**	**% BM**	**Biopsy Slte**	**Tissue**	**DNA/RNA**	**Method 1**	**MYD88 L265P**	**Method 2**	**MYD88 L265P**	**Final MYD88 L265P**
1	WM	m	62	1.14	20	BM	$CD19^+$/$CD138^+$	DNA	Exome	L265P	ND	ND	L265P
2	WM	f	78	2	80	BM	$CD19^+$/$CD138^+$	DNA	Exome	L265P	ND	ND	L265P
3	WM	m	67	5.1	50	BM	$CD19^+$/$CD138^+$	DNA	Exome	L265P	ND	ND	L265P
4	WM	m	73	3.8	50	BM	$CD19^+$/$CD138^+$	DNA	Exome	L265P	ND	ND	L265P
5	WM	m	76	3.6	50	BM	$CD19^+$/$CD138^+$	DNA	Exome	WT	ND	ND	WT
6	WM	m	79	1.9	70	BM	$CD19^+$/$CD138^+$	DNA	Exome	L265P	ND	ND	L265P
7	WM	m	51	1.9	50	BM	$CD19^+$/$CD138^+$	DNA	Exome	L265P	ND	ND	L265P
8	WM	m	75	1.6	60	BM	$CD19^+$/$CD138^+$	RNA	Sanger	L265P	ND	ND	L265P
9	WM	m	79	3.4	70	BM	$CD19^+$/$CD138^+$	RNA	Sanger	L265P	ND	ND	L265P
10	WM	m	59	3.2	60	BM	$CD19^+$/$CD138^+$	RNA	Sanger	L265P	ND	ND	L265P
11	WM	m	71	1.5	40	BM	$CD19^+$/$CD138^+$	DNA	Sanger	L265P	ASO-PCR	L265P	L265P
12	WM	m	69	0.3	5	BM	$CD19^+$/$CD138^+$	RNA	Sanger	L265P	ND	ND	L265P
13	WM	m	64	2.7	60	BM	$CD19^+$/$CD138^+$	DNA	Sanger	WT	ASO-PCR	L265P	L265P
14	WM	m	64	3	90	BM	$CD19^+$/$CD138^+$	RNA	Sanger	L265P	ND	ND	L265P
15	WM	m	66	2.5	10	BM	$CD19^+$/$CD138^+$	DNA	Sanger	L265P	ASO-PCR	L265P	L265P
16	WM	m	54	1.2	70	BM	$CD19^+$/$CD138^+$	DNA	Sanger	WT	ASO-PCR	L265P	L265P
17	WM	m	71	1.3	80	BM	$CD19^+$/$CD138^+$	DNA	Sanger	L265P	ND	ND	L265P
18	WM	m	86	1.8	60	BM	$CD19^+$/$CD138^+$	DNA	Sanger	L265P	ASO-PCR	L265P	L265P
19	WM	m	43	4.2	50	BM	$CD19^+$/$CD138^+$	DNA	Sanger	L265P	ASO-PCR	L265P	L265P
20	WM	m	76	2.1	80	BM	$CD19^+$/$CD138^+$	DNA	Sanger	L265P	ASO-PCR	L265P	L265P
21	WM	m	50	0.7	20	BM	$CD19^+$/$CD138^+$	DNA	Sanger	L265P	ND	ND	L265P
22	WM	m	68	2.8	20	BM	$CD19^+$/$CD138^+$	DNA	Sanger	WT	ASO-PCR	L265P	L265P
23	WM	m	71	2	50	BM	$CD19^+$/$CD138^+$	DNA	Sanger	L265P	ASO-PCR	L265P	L265P
24	WM	m	52	2.1	50	BM	$CD19^+$/$CD138^+$	RNA	Sanger	L265P	ND	ND	L265P
25	WM	m	70	1.3	20	BM	$CD19^+$/$CD138^+$	RNA	Sanger	L265P	ND	ND	L265P
26	WM	f	67	1.3	15	BM	$CD19^+$/$CD138^+$	RNA	Sanger	L265P	ND	ND	L265P
27	WM	m	71	3.8	95	BM	$CD19^+$/$CD138^+$	RNA	Sanger	L265P	ND	ND	L265P
28	WM	m	80	0.7	5	BM	$CD19^+$/$CD138^+$	RNA	Sanger	L265P	ND	ND	L265P
29	WM	m	79	1.5	10	BM	$CD19^+$/$CD138^+$	DNA	Sanger	L265P	ASO-PCR	L265P	L265P
30	WM	m	55	4.2	70	BM	Unsorted	DNA	Sanger	WT	ASO-PCR	L265P	L265P
31	WM	f	73	2.7	40	BM	$CD19^+$/$CD138^+$	DNA	Sanger	L265P	ASO-PCR	L265P	L265P
32	WM	f	61	1.65	40	BM	Unsorted	DNA	Sanger	WT	ASO-PCR	L265P	L265P
33	WM	f	73	0.4	50	BM	$CD19^+$/$CD138^+$	DNA	Sanger	L265P	ASO-PCR	L265P	L265P
24	WM	m	59	1.9	50	BM	Unsorted	DNA	Sanger	L265P	ASO-PCR	L265P	L265P
35	WM	m	67	2.4	50	LN	Unsorted	DNA	Sanger	L265P	ASO-PCR	L265P	L265P
36	WM	m	78	2.1	70	LN	Unsorted	DNA	Sanger	L265P	ASO-PCR	L265P	L265P
37	WM	m	59	3.7	35	LN	Unsorted	DNA	Sanger	L265P	ASO-PCR	L265P	L265P
38	WM	m	62	4.2	ND	LN	Unsorted	DNA	Sanger	L265P	ASO-PCR	L265P	L265P
39	WM	m	50	1.3	30	LN	Unsorted	DNA	Sanger	L265P	ASO-PCR	L265P	L265P
40	LPL	m	61	4.57	5	LN	Unsorted	DNA	Sanger	WT	ASO-PCR	WT	WT
41	LPL	m	62	0.2	50	LN	Unsorted	DNA	Sanger	WT	ASO-PCR	WT	WT
42	LPL	f	48	1.3	0	LN	Unsorted	DNA	Sanger	WT	ASO-PCR	WT	WT
43	LPL	m	58	small IgM	40	LN	Unsorted	DNA	Sanger	WT	ASO-PCR	WT	WT
44	LPL	f	73	0	20	LN	Unsorted	DNA	Sanger	WT	ASO-PCR	WT	WT

IgM is reported in g/dL. Percent bone marrow involvement (% BM), bone marrow (BM), lymph node (LN), analysis not done (ND).
