# Supplementary material for: Activation of TAK1 by MYD88 L265P drives malignant B-cell Growth in non-Hodgkin lymphoma
Source: Blood Cancer J. 2014 Feb 14;4(2):e183–. doi: 10.1038/bcj.2014.4 (PMC3944662; doi:10.1038/bcj.2014.4)
Supplement: Supplementary Table 2 [file bcj20144x3.pdf]

Gene	Chromosome	Start Position	Type	Reference	Variant	N
**IGSF21**	chr1	18692038	cSNV	C	T	1
**UBR4**	chr1	19499575	cSNV	A	G	1
**HENMT1**	chr1	109192963	cSNV	C	T	1
**SPAG17**	chr1	118634339	cSNV	T	A	1
**SPAG17**	chr1	118644468	cSNV	G	A	1
**NOS1AP**	chr1	162336895	cSNV	G	A	1
**GNPAT**	chr1	231408084	INDEL	C	CG	1
**GNPAT**	chr1	231408087	INDEL	C	CAA	1
**FMN2**	chr1	240256620	cSNV	G	A	1
**ST6GAL2**	chr2	107446684	cSNV	G	T	1
**FSIP2**	chr2	186653958	cSNV	T	G	1
**SPHKAP**	chr2	228881615	cSNV	G	A	1
**NDUFA10**	chr2	240954206	cSNV	T	C	1
**TOP2B**	chr3	25661473	cSNV	A	T	1
**MYD88**	chr3	38182641	cSNV	T	C	4
**FGG**	chr4	155529715	cSNV	A	C	1
**GHR**	chr5	42719285	cSNV	G	A	1
**CCL28**	chr5	43381991	cSNV	C	T	1
**FSTL4**	chr5	132535160	cSNV	C	T	1
**DST**	chr6	56401696	cSNV	G	A	1
**DNAH11**	chr7	21750239	cSNV	A	T	1
**ZAN**	chr7	100350553	cSNV	C	A	1
**POT1**	chr7	124511089	cSNV	C	T	1
**ZNF704**	chr8	81577286	cSNV	C	G	1
**NUDCD1**	chr8	110308630	cSNV	C	A	1
**C9orf79**	chr9	90501029	INDEL	AG	A	1
**NRP1**	chr10	33545304	cSNV	C	T	1
**A1CF**	chr10	52601523	INDEL	C	CT	1
**C10orf131**	chr10	97697788	cSNV	G	A	1
**OR5F1**	chr11	55761425	cSNV	A	G	1
**ANAPC5**	chr12	121784690	INDEL	GT	G	1
**PIWIL1**	chr12	130840190	cSNV	A	T	1
**BRF1**	chr14	105677470	cSNV	A	T	1
**TP53BP1**	chr15	43701243	cSNV	G	A	1
**TP53**	chr17	7578289	cSNV	C	A	1
**RAB8A**	chr19	16243055	cSNV	G	A	1
**MLL4**	chr19	36211579	cSNV	C	T	1
**ATP1A3**	chr19	42482455	cSNV	C	T	1
**MEIS3**	chr19	47910395	cSNV	C	A	1
**LENG8**	chr19	54969313	cSNV	C	T	1
**WFDC10A**	chr20	44259602	cSNV	C	A	1
**GNAS**	chr20	57484783	cSNV	A	G	1
**NYX**	chrX	41333458	cSNV	G	A	1
**DUSP21**	chrX	44703608	cSNV	G	A	1

Supplementary Table 2. Somatic cSNV and INDELs detected in paired tumor-normal samples from patients with WM (n=5). cSNVs were included on this list after filtering on the following parameters: total read depth in both normal and tumor samples >40, alternative allele read depth in normal samples <10, alternative allele read depth in tumor samples >20 and variant classification of

non-sense, frame-shifting, splice site or missense. INDELs were filtered by a total read depth >20. Ref, reference allele; variant, variant allele.
