# Supplementary material for: Activation of TAK1 by MYD88 L265P drives malignant B-cell Growth in non-Hodgkin lymphoma
Source: Blood Cancer J. 2014 Feb 14;4(2):e183–. doi: 10.1038/bcj.2014.4 (PMC3944662; doi:10.1038/bcj.2014.4)
Supplement: Supplementary Table 3 [file bcj20144x4.pdf]

Chromosome	Cytoband	CNA	Size (Mb)	Sample ID#
1	p31.3-p22.2	loss	30	17
1	p36.13-p35.3	loss	10	2
3	3*	gain	146	17
6	q16.3-q27*	loss	64	19
6	p25.3-p21.1*	gain	42	34
6	p21.1-q27*	loss	129	34
6	p25.3*-p11.2	gain	58	2
6	q11.1-q27*	loss	109	2
6	q16.3-q27*	loss	69	14
7	7	gain	159	2
8	p23.3-p23.1	loss	7	34
8	p23.1-p22	gain	8	34
8	p22-p21.2	loss	12	34
8	p21.2-q24.3	gain	119	34
8	p23.3-p12	loss	29	22
9	9	gain	141	34
10	10	gain	135	17
11	p15.5-p14.3	gain	23	4
11	q14.3-q25*	loss	44	4
11	q24.1-q25*	loss	13	17
12	12	gain	133	22
13	13	loss	115	17
13	q14.1-q21.1*	loss	15	22
15	15	gain	102	17
17	p13.3-p11.2*	loss	20	2
17	q21.32-q25.3	gain	36	14
18	18*	gain	78	17
21	21	loss	48	22

*Previously detected in WM.

Supplementary Table 3. Copy number abnormalities (CNAs) predicted by mate pair analysis in WM (n=15). The location of predicted deletions and amplifications of chromosomal regions greater than 10 Mb in size were determined from count plots as described in the Methods section.
