# Supplementary material for: Activation of TAK1 by MYD88 L265P drives malignant B-cell Growth in non-Hodgkin lymphoma
Source: Blood Cancer J. 2014 Feb 14;4(2):e183–. doi: 10.1038/bcj.2014.4 (PMC3944662; doi:10.1038/bcj.2014.4)
Supplement: Supplementary Methods [file bcj20144x5.pdf]

Supplementary Methods

Exome Sequencing

Sequencing reads were aligned to the Hg19 reference genome using Novalign algorithm (Novocraft, 2010), GATK was applied for base quality score recalibration and indel realignment, and Picard was used for duplicate marking (1). Somatic mutations were called using SomaticSniper for the five paired samples and GATK UnifiedGenotyper for the SNV and INDEL calling for the two unpaired tumor samples (2, 3). Mutations were annotated using public databases using the TREAT pipeline (4). The annotations include, but are not limited to, information from dbsnp135, 1000genomes, ESP5400, COSMIC and functional impact predictions by SIFT and SNPEFF (5-7). Several post-processing filters were applied to remove false positive variants to achieve high specificity. The somatic SNV variants were first filtered by total read depth (>40 in normal and tumor samples) and then further filtered by alternative allele read depth in normal samples (<10) and in tumor samples (>/= 40). INDEL variants were filtered only by total read depth (>20) due to the overall low number of reads supporting these variants.

Due to the potential heterogeneity of tumor samples obtained by $CD19^{+}CD138^{+}$ sorting of patient bone marrow cells, mean absolute deviation (MAD) scores were calculated to ensure that adequate differences in tumor content existed between the tumor and control samples. For each heterozygous variant, the ratio of reads corresponding to both the reference and non-reference alleles was calculated. In non-tumor samples, the ratio for heterozygotes should

be close to 50%. However, ratios show greater variance in tumor samples due to tumor clonality, tumor content, copy gains, copy losses and loss of heterozygosity. Ratios for both tumor and control samples were determined with the circular binary segmentation algorithm used in the DNAcopy R package, and the MAD score calculated by comparing these ratios with the expected ratio for a heterozygous variant in a control sample (50%). All tumor samples had markedly higher MAD scores than the corresponding controls, indicating that despite potential differences in the percentage of tumor cells obtained from patient bone marrows, all tumor samples contained significantly more tumor tissue than their respective controls. To look for structural abnormalities in the matched patient samples, the ratios of the tumor and matching control MAD scores were calculated. As alleles can be both lost and gained, the absolute value of the $\log_2$ transformation was used. These values were then plotted separately for each chromosome with differences from baseline indicating a genetic imbalance between the tumor and the control sample.

Supplementary References

1. McKenna A, Hanna M, Banks E, Sivachenko A, Cibulskis K, Kernytsky A, *et al.* The Genome Analysis Toolkit: a MapReduce framework for analyzing next-generation DNA sequencing data. *Genome Res* 2010 Sep; **20**(9)**:** 1297-1303.

2. Larson DE, Harris CC, Chen K, Koboldt DC, Abbott TE, Dooling DJ, *et al.* SomaticSniper: identification of somatic point mutations in whole genome sequencing data. *Bioinformatics* 2012 Feb 1; **28**(3)**:** 311-317.

3. DePristo MA, Banks E, Poplin R, Garimella KV, Maguire JR, Hartl C*, et al.* A framework for variation discovery and genotyping using next-generation DNA sequencing data. *Nature genetics* 2011 May; **43**(5)**:** 491-498.

4. Asmann YW, Middha S, Hossain A, Baheti S, Li Y, Chai HS*, et al.* TREAT: a bioinformatics tool for variant annotations and visualizations in targeted and exome sequencing data. *Bioinformatics* 2012 Jan 15; **28**(2)**:** 277-278.

5. Cingolani P, Platts A, Wang le L, Coon M, Nguyen T, Wang L*, et al.* A program for annotating and predicting the effects of single nucleotide polymorphisms, SnpEff: SNPs in the genome of Drosophila melanogaster strain w1118; iso-2; iso-3. *Fly* 2012 Apr-Jun; **6**(2)**:** 80-92.

6. Kumar P, Henikoff S, Ng PC. Predicting the effects of coding non-synonymous variants on protein function using the SIFT algorithm. *Nature protocols* 2009; **4**(7)**:** 1073-1081.

7. Ng PC, Henikoff S. SIFT: Predicting amino acid changes that affect protein function. *Nucleic acids research* 2003 Jul 1; **31**(13)**:** 3812-3814.
